# Cell Proteomic Footprinting: Advances in the Quality of Cellular and Cell-Derived Cancer Vaccines

**DOI:** 10.3390/pharmaceutics15020661

**Published:** 2023-02-16

**Authors:** Petr G. Lokhov, Elena E. Balashova, Oxana P. Trifonova, Dmitry L. Maslov, Alexander I. Archakov

**Affiliations:** Institute of Biomedical Chemistry, 10 Building 8, Pogodinskaya Street, 119121 Moscow, Russia

**Keywords:** proteomic footprint, cancer vaccine, antigens, vaccination, cell authentication, quality control, mass spectrometry

## Abstract

In omics sciences, many compounds are measured simultaneously in a sample in a single run. Such analytical performance opens up prospects for improving cellular cancer vaccines and other cell-based immunotherapeutics. This article provides an overview of proteomics technology, known as cell proteomic footprinting. The molecular phenotype of cells is highly variable, and their antigenic profile is affected by many factors, including cell isolation from the tissue, cell cultivation conditions, and storage procedures. This makes the therapeutic properties of cells, including those used in vaccines, unpredictable. Cell proteomic footprinting makes it possible to obtain controlled cell products. Namely, this technology facilitates the cell authentication and quality control of cells regarding their molecular phenotype, which is directly connected with the antigenic properties of cell products. Protocols for cell proteomic footprinting with their crucial moments, footprint processing, and recommendations for the implementation of this technology are described in this paper. The provided footprints in this paper and program source code for their processing contribute to the fast implementation of this technology in the development and manufacturing of cell-based immunotherapeutics.

## 1. Introduction

The development of new immunotherapeutics is one of the important directions in improving cancer treatment [1]. Since cancers are diverse and often arise suddenly, treatment must be both specific and flexible, and ideally long-lasting. Vaccination may offer such treatment [2]. The widely accepted idea that you should vaccinate with what you wish to acquire immunity against was put into practice with the development of the first cancer vaccines, which were based on inactivated cancer cells. Numerous vaccines of this nature have been developed against a significant number of cancers, including melanoma [3,4,5], lung cancer [6,7,8], prostate cancer [9], colorectal cancer [10,11,12,13], and renal cell cancer [14,15,16]. Many vaccines were tested in clinical trials, but none have yet passed the last stage [17,18].

Although vaccines based on cancer cells have failed in clinical trials, research in this area remains at a high level [19]. The reason for this is a straight immunological basis with clear mechanism of action, and cancer cells are a fully fledged source of an entire variety of native antigens and persistent drivers behind the development of new cellular cancer vaccines [20]. The lack of effectiveness of cancer vaccines, which prevents their implementation, is being compensated by the use of combinatorial therapy, the use of adjuvants [21,22], cell modification (leading to an increase in their immunogenicity) [23], the use of checkpoint inhibitors [24,25], etc. However, an assessment of cancer cells based on their antigenic composition, which is close to the body’s cells and is changeable, has not been examined in detail.

It is known that the cultivation of mammalian cells, which is a common step in the manufacture of cell-based products, leads to cellular degeneration accompanied by modifications in their cell surface molecular profile [26,27]. One of the reasons for this is that the genomic instability characteristic of cancer cells causes their pronounced heterogeneity at the genome level, which is reflected in their molecular profiles. It is assumed that these changes in cell culture are exacerbated by in vitro selection pressure [28], which subsequently leads to the divergence of cellular molecular phenotypes over time. Because most existing cell lines have already been passaged multiple times, significant variation has already been introduced into their antigen expression patterns [29,30]. Moreover, even cell lines derived from the same specimen and cultured for a relatively brief period displayed marked differences [28], which indicates the appearance of pronounced heterogeneity at the post-genome level.

Unfortunately, the design and manufacture of cell products are limited to cell authentication and possibly an assessment of the expression of several individual antigens. With regards to the current state of life sciences, it seems insufficient, especially in light of the existence of high-throughput molecular profiling methods widely used in omics sciences. This article is devoted to a review of one of such methods, known as ‘cell proteomic footprinting’ (CPF) [31], which makes it possible to ensure that cellular cancer vaccines and other cell-based immunotherapeutics are produced from exactly the right cells in the right way.

## 2. Background of Cell Proteomic Footprinting

Many molecules within a cell are associated with basic cellular functions such as biosynthesis, energy balance, and cell division, and the antigenic determinants associated with the molecules involved are mostly common to all cells in the body [32,33,34]. The surface of the cell is the place where its individuality is most expressed. A variety of environmental factors, including contact with neighboring cells, contact with surrounding structures, and the presence of specific receptors, contribute to the unique ‘face’ of each cell type [35,36,37]. One clear example of this is that when cancer cells appear in the body, the immune system recognizes and removes them, even if the molecular phenotype of the cancer cells is similar to that of normal cells. The antibodies and killer cells sent by the immune system only recognize the antigens that are present on the surface of cancer cells, as they do not penetrate inside a living cell. This fact demonstrates that the cell surface is sufficient for the accurate recognition of cells and their precise differentiation from each other [36].

**Figure 1:**
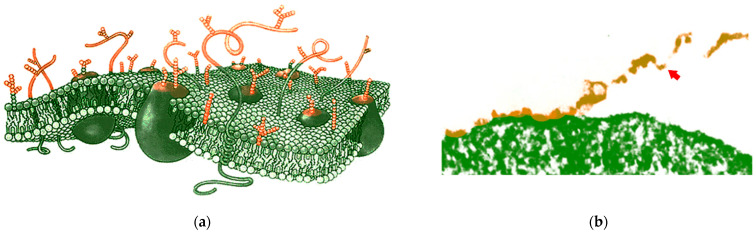
Cell surface molecules and isolation of cell surface material. (**a**) Schematic three-dimensional cross-section of a cell membrane. Proteins whose extracellular fragments (more or less glycosylated) are exposed outside the cell (marked in color) and form the antigenic properties of the cell. These extracellular protein parts can be detached from the cell by treating them with trypsin. Adapted from [40]. (**b**) Microphotography of the partial detachment of aggregated cell surface material (indicated with orange color and an arrow) following treatment of the cell with 0.2% trypsin. Magnification, 19,000×. Adapted from [38].

Therefore, in order to develop immunotherapeutic agents that are effective against cancer cells, such as cancer vaccines, it is necessary to ‘read’ the ‘face’ of cancer target cells to ensure that the vaccine has the correct antigen composition. The bulk of specific molecules on the cell surface are proteins and, associated with them, carbohydrates (Figure 1a). To ‘read’ the cell surface, it must first be isolated. With this, it is necessary to treat living cells with a protease, e.g., trypsin. Since the protease is quite large, it does not penetrate the cell and therefore only cleaves the proteins that are present on the surface (Figure 1b) [38,39].

This represents a starting point for the development of CPF. Trypsin occupies a central role in proteomics—an omics science and a technology platform used for the high-throughput analysis of proteins in biological samples [41,42]. The meaning of trypsin use in proteomics is that it cleaves proteins into fragments at specific sites [43]. The occurrence of these sites in proteins is such that a number of cleaved fragments is enough to obtain a protein-specific picture. At the same time, the number of amino acids in these fragments is such that they have a specific composition and, accordingly, molecular weights that make it possible to identify proteins by mass spectrometry (the so-called peptide fingerprinting [44]).

Proteomics science has evolved over time, resulting in perfected proteome analysis protocols, the availability of required pure proteomics-grade reagents (including proteomics-grade trypsin), and experience in proteomic data processing [45]. All of this established the background for the emergence of CPF [46], which is essentially the use of trypsin to treat the surface of cells to obtain a specific proteomic pattern consisting of trypsinolytic fragments of cell surface proteins. Since this pattern is specific to cells, it enables them to be identified and authenticated, confirming that they have expected therapeutic properties.

CPF is largely relevant in the development and manufacture of whole-cell, cell lysate, cell membrane, and antigenic essence-based vaccines that utilize cancer cells and cells from the tumor microenvironment, such as microvascular endothelial cells and fibroblasts. A recent review discusses the application of technology in the development of a variety of cancer vaccines based on the antigenic essence of cells [47]. The list of cancer vaccines developed using CPF is still limited by the antiangiogenic vaccine [48]. This is due to the fact that the CPF test is a relatively new direction in the field of cancer vaccines, which inspired the writing of this analytical review.

## 3. Cell Proteomic Footprinting

**Figure 2:**
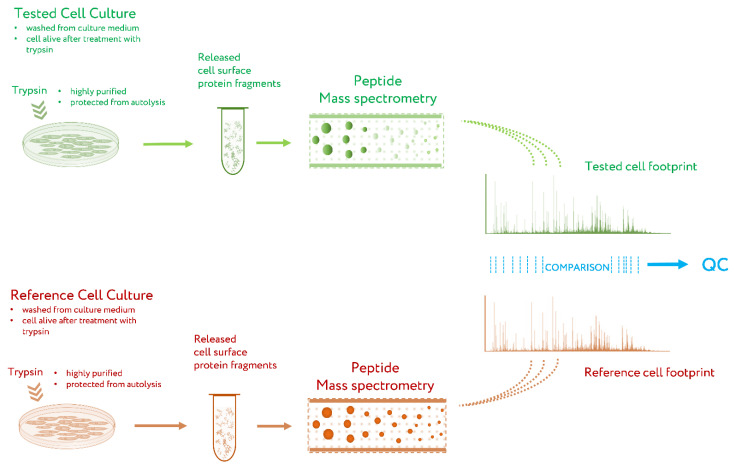
Workflow of cell proteomic footprinting. QC, quality control. Adapted from [47].

The general workflow for CPF is presented in Figure 2 [47]. Briefly, the cell culture to be tested by CPF is washed several times to remove traces of the culture medium, especially the proteins that can be absorbed on the cell surface. After this, the cells are treated with a solution of proteomics-grade trypsin [49] under mild conditions, so that the cells after treatment are alive but extracellular fragments of proteins are cleaved off [39]. The solution with cleaved protein fragments is taken, and a sample of it is subjected to peptide mass spectrometry. The obtained list of peptide masses is a cell proteomic footprint that is specific to the tested cell culture. By comparing this footprint with a reference footprint corresponding to the cells with the desired properties, the presence of these properties, their absence, or the degree of deviation from them is evaluated for the tested cell culture [47].

## 4. Cell Authentication by Cell Proteomic Footprinting

Cross-contamination between cell lines was found to be a common problem in studies of cultivated cells [50,51,52,53,54,55]. It was shown that up to 36% of cell lines appear to have a different origin than their initial cell lines [54,56]. Moreover, even the most popular cell line, HepG2, which is widely used in scientific research and common in laboratories, raises questions due to its heterogeneity [57]. Today, a method of cell authentication based on relatively stable genotype data, short tandem repeat (STR) markers (tiny repetitive segments of DNA found between genes), is widely used [58,59,60,61,62].

However, for cell products, it is important to not only authenticate the cell type at the genome level. For example, an established cell culture can multiply many times and exhibit characteristic properties, including therapeutic ones. However, close to senescence, the primary culture degenerates and begins to die [63,64,65,66,67], while the genotype (both at the beginning and at senescence) is the same. Therefore, authentication at the molecular phenotype level (post-genome level) is also needed for the development of cell products. Thus, CPF, relating to the molecular phenotype associated with the confirmation of the therapeutic qualities of cells, complements genotyping via the STR assay.

**Figure 3:**
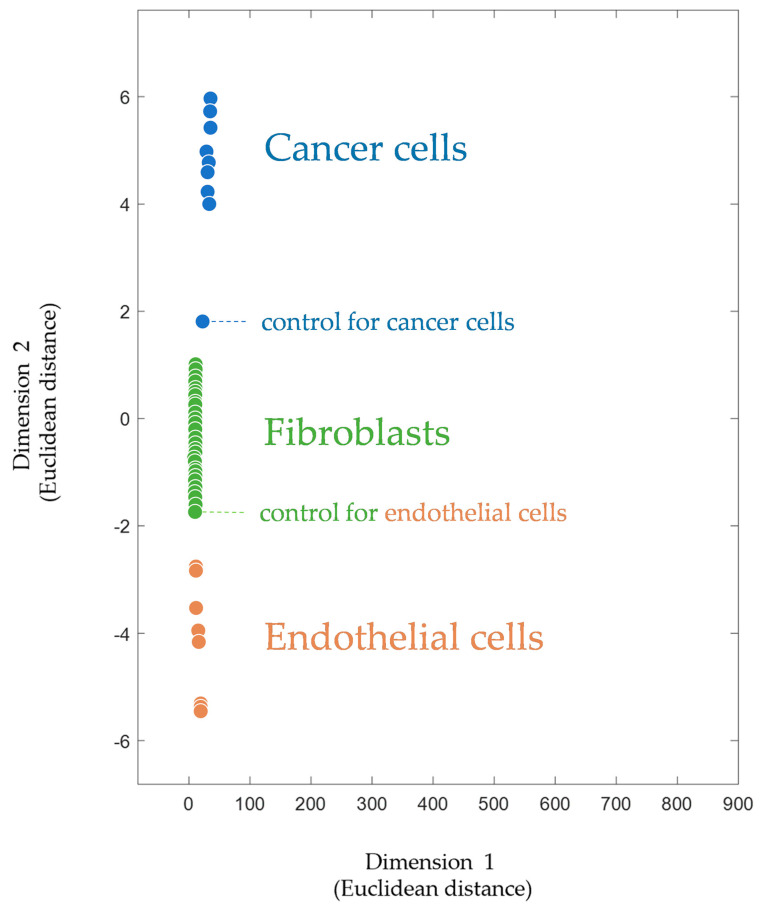
Multidimensionally scaled cell proteomic footprints of various cell types. Footprints of endothelial cells (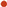) are taken from [31] and correspond to primary cultures of human microvascular endothelial cells (HMECs) with native and tumor-induced phenotypes and from different donors. Footprints of fibroblasts (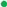) are taken from [46] and correspond to primary cultures of human fibroblasts with different origins. Footprints of cancer cells (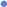) are taken from [80] and correspond to different cancer cell lines, both untreated and drug-treated. The footprints for this plot as well as the script (for Matlab software, MathWorks, Natick, MA, USA) for their processing are provided in the Supplementary Materials.

It is worth noting that subjecting trypsinolytic protein fragments to mass spectrometry for cell identification is not new and has been widely used. Matrix-assisted laser desorption ionization–time of flight mass spectrometry (MALDI-TOF MS) is increasingly used in diagnostic bacteriology. Well-established commercial systems allow for the fast and reliable identification of bacteria [68,69,70]. Thus, the MALDI Biotyper (Bruker Daltonik GmbH, Billerica, MA, USA) [71] identifies germs via the peptide fingerprinting of proteins [72,73,74]. The use of CPF is similar to that of Biotyper, but for mammalian cells. Figure 3 shows how CPF clearly divides mammalian cells into groups according to their type. Since the footprint is a multivariable (multidimensional) characteristic, multidimensional scaling is applied for the visualization of footprints in a two-dimensional plane. In addition human cancer cells, endothelial cells (human microvascular endothelial cells, HMECs), and fibroblasts of various origins are represented. These cell types, like cancer cells themselves, are also considered targets for immunotherapy, as they are part of the tumor microenvironment and are required to ensure its functioning [75,76,77,78,79].

Although the intensity of the mass peaks in the proteomic footprint largely depends on the protocols used for sample preparation and mass spectrometry, the composition of the footprint is objective since it is determined by fragments of cell-specific proteins. Thus, binary-encoded footprints obtained in different years and on different mass spectrometers (but of the same type) are consistent with each other. The footprint of fibroblasts used as a control sample in the footprinting experiments of endothelial cells [31] belongs to the group of fibroblast footprints obtained in another study [46] (Figure 3).

The proof that CPF can objectively identify and authenticate cell types with high accuracy is presented in Table 1.

The cell authentication with high efficiency presented in Table 1 is more conceptual than practical. As mentioned above, the generally accepted STR-based assay copes with this task quite well. However, the authentication of subpopulations of cancer cells, as well as subpopulations of cells related to the tumor microenvironment, such as HMECs and fibroblasts, with a distinctive molecular phenotype and therapeutic properties that are not available for STR, relates to the direct application of CPF. Evidence that CPF can objectively identify and authenticate subpopulations of cells with different therapeutic properties is presented in Table 2.

Fibroblast cultures are a very good model to demonstrate CPF efficacy in relation to the authentication of cell subtypes because different subtypes of fibroblasts have identical morphologies and are propagated under the same conditions. Moreover, cancer-associated fibroblasts (CAFs), like normal fibroblasts, constitute a heterogenous population of cells from different origins [81]. CAFs actively participate in the regulation of different biological cancer behaviors such as carcinogenesis [82], metastasis [83], and therapy resistance [84]. Therefore, fibroblast subpopulations represent promising targets for cancer immunotherapy [76,77,78,85]. Footprints for three different subpopulations of human fibroblasts from the previously published studies [46] were compared: primary cultures of dermal papilla fibroblasts (*n* = 17), adipose-derived fibroblasts (*n* = 4), and skin fibroblasts (*n* = 4). All fibroblasts have identical spindle-like morphologies in cultures but exhibit different therapeutic properties according to their origin. Dermal papilla fibroblasts are trichogen cells [86], adipose-derived fibroblasts are pluripotent [87], and adult skin fibroblasts are better for autologous cell therapy [88]. Table 2 demonstrates the high efficacy of separating the footprints of fibroblast subpopulations. Thus, CPF can be recognized as a tool to authenticate cell cultures at the subpopulation level, confirming their therapeutic properties.

**Figure 4:**
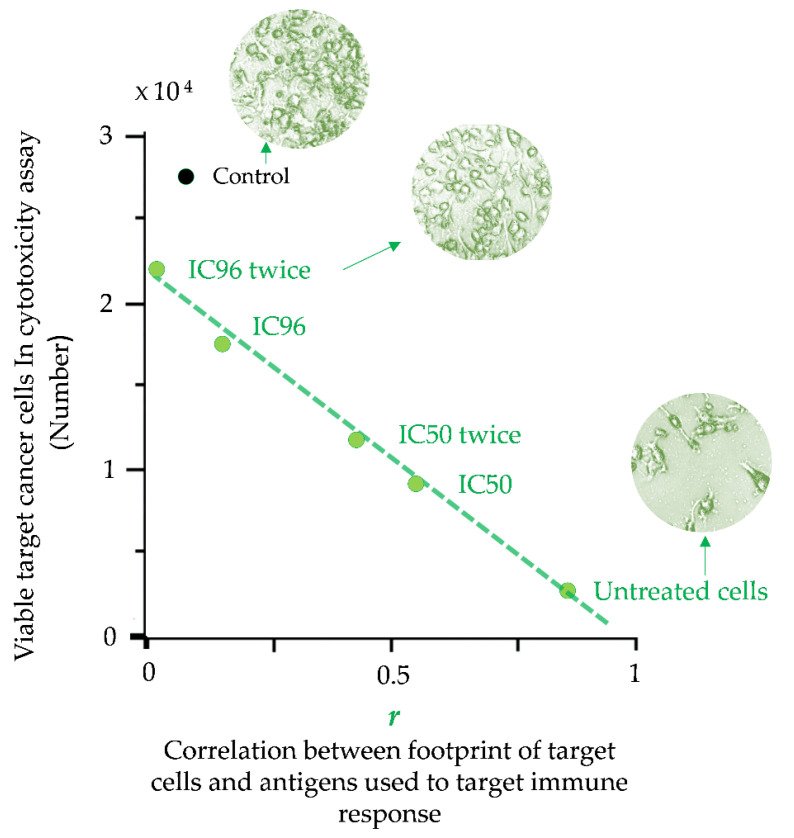
Escape of target cancer cells from the immune response in the cytotoxicity assay (CTA) as a result of their surface profile changes induced by the drug treatment. Points are presented for untreated MCF-7 target cells or cells treated with a single IC96 or IC50 dose of etoposide or two separate IC96 or IC50 doses (‘twice’) of etoposide (adapted from [80]). A linear approximation is shown. The average number of viable cells for the 3 experiments is presented. Correlation coefficients (*r*) were calculated for the correlation between the antigenic composition used to induce an immune response in CTA (known as cellular antigenic essence [89]) and the proteomic footprints of target cancer cells. ‘Control’ corresponds to the immune response induced by the control antigen composition (non-relevant to target MCF-7 cells). The data in the graph show that the escape of target cells from the immune response is directly related to the degree of change in their proteomic footprint.

A footprint is an accurate multi-parameter characteristic of cells that can also be directly related to the therapeutic properties of vaccines based on cancer cells. The direct connection between the footprint of cancer cells and their escape from the immune response was demonstrated in cytotoxicity assays (CTAs) [80]. The molecular phenotype of target cancer cells (MCF-7) was gradually changed under drug-induced cell selection pressure. Results from the CTA show that the rate of cell escape from the immune response depends on the similarity between the antigen composition (cellular antigenic essence) used to induce an immune response and the footprint of target cancer cells (Figure 4) [80]. These results also demonstrate that the set of antigens expressed on the surface of cancer cells can be significantly altered under drug treatment, and such alterations create cell subpopulations that completely evade the immune response.

Moreover, the results in Table 2 demonstrate that both the strength of drug treatment (IC50 versus IC96) and also the type of drug (etoposide versus doxorubicin and tamoxifen) inform the appearance of a subpopulation (with an area under ROC curve (AUC) = 0.99) of cancer cells in terms of their surface molecular profile. This strong point suggests that the antigen composition for cancer vaccine should be designed to account for the cell footprints.

Endothelial cells (ECs) line the inner surface of blood vessels and constitute a selective barrier between the blood and the tissue. The importance of ECs in the context of cancer has been extensively investigated [90]. In 1945, it was reported that a tumor recruits microvasculature from surrounding tissues to support its feeding and growth [91]. This finding yielded an entire field of research that aims to inhibit new blood vessel formation [90]. Vaccination against EC in tumors offers the additional benefit that ECs are genetically more stable and therefore less likely to develop escape mutations than cancer cells [92]. Moreover, the ratio of ECs to cancer cells in tumors is approximately 1:100. The destruction of a small number of ECs can lead to vascular obstruction and arrest tumor growth because vascular integrity is essential to tumor feeding [93,94,95,96].

The molecular phenotype of ECs in the microvasculature is tissue-specific [97,98], and if the microvasculature is involved in tumor feeding, the EC phenotype also becomes tumor-specific [99,100,101]. This heterogeneity in the phenotype provides grounds for designing antiangiogenic vaccines for anti-cancer vaccination. The influence of tumors on the EC surface profile was investigated by culturing HMECs with a tumor-conditioned medium [31]. CPF demonstrated that cancer cells induce HMEC subpopulations with statistically significant phenotypic differences from the initial cells (Table 2) that are directly connected with their escape from the immune system. This finding provides a strong justification for designing and manufacturing a cancer vaccine that targets tumor vessels under CPF control.

## 5. Cell Proteomic Footprinting Protocols

### 5.1. Sample Preparation

The sample preparation protocol used in the reviewed CPF studies is presented below. This protocol has been validated for cancer cells, endothelial cells, and fibroblasts, but can be adapted to other cells as well. For example, the conditions for washing and treating cells with trypsin can be chosen so as not to cause cell destruction, but rather to obtain a significant amount of cell surface content.

Protocol 1: Sample preparation (adapted from [31,46,80])

1. Cells grown to 65% confluence are washed four times with Hanks’ balanced salt solution (HBSS). During the third and fourth washings, the cells are kept in HBSS for 15 min. Washed cells are treated with 0.2 μg/mL of trypsin (15,000 U/mg, Promega, Madison, WI, USA) in HBSS. A 0.5 mL trypsin solution is added to every 10 cm^2^ of a cell culture plate, incubated for 20 min at 37 °C in saturated humidity, and then the trypsin solution is carefully collected (to avoid the detachment of cells and their entry into the collected trypsin solution).
2. The collected trypsin solution is centrifuged (600× *g* for 5 min) to remove detached cells (if any, cells mainly remain attached during and after treatment with trypsin; however, there should not be a time lag between cell treatment with trypsin and this step to minimize the damage of detached cells).
3. The collected trypsin solution is acidified (up to pH 4) by adding 10% acetic acid to stop trypsin action (a litmus test is used to confirm pH).
4. The resulting solution contains cell surface antigens. Cells just after treatment with trypsin are covered by HBSS with 10% fetal bovine serum (FBS), and their viability is estimated by trypan blue exclusion or another suitable method [102]. The number of dead cells should not exceed 1%.

The presence of protein fragments originating from the culture medium in the analyzed samples is sufficiently reduced by washing the cells four times before trypsin treatment. The use of highly purified proteomics-grade trypsin is mandatory to avoid (i) the contamination of obtained cell surface antigens with impurities of the trypsin preparation, (ii) cell death during trypsin treatment that is pronounced in the case of using unpurified trypsin [38]; (iii) the contamination of cell surface antigens with trypsin autolysis products (proteomics-grade trypsin is protected from autolysis).

Samples should be stored at −20 °C or lower by default. Since samples are peptide compositions, though this storage temperature is optimal. However, for storage (several months to years) in the longer term, it is more preferable to store peptides in a freezer at −80 °C. Freeze–thaw cycles should be avoided by freezing individual aliquots.

### 5.2. Mass Spectrometry

Mass spectrometry is an effective method for assessing the composition of peptide solutions to which obtained samples are related. Although there are many effective protocols for peptide mass spectrometry, it is reasonable to provide one in this review that has already been tested in several CPF studies.

Protocol 2: Mass spectrometry analysis (adapted from [31,46,80])

**Figure 5:**
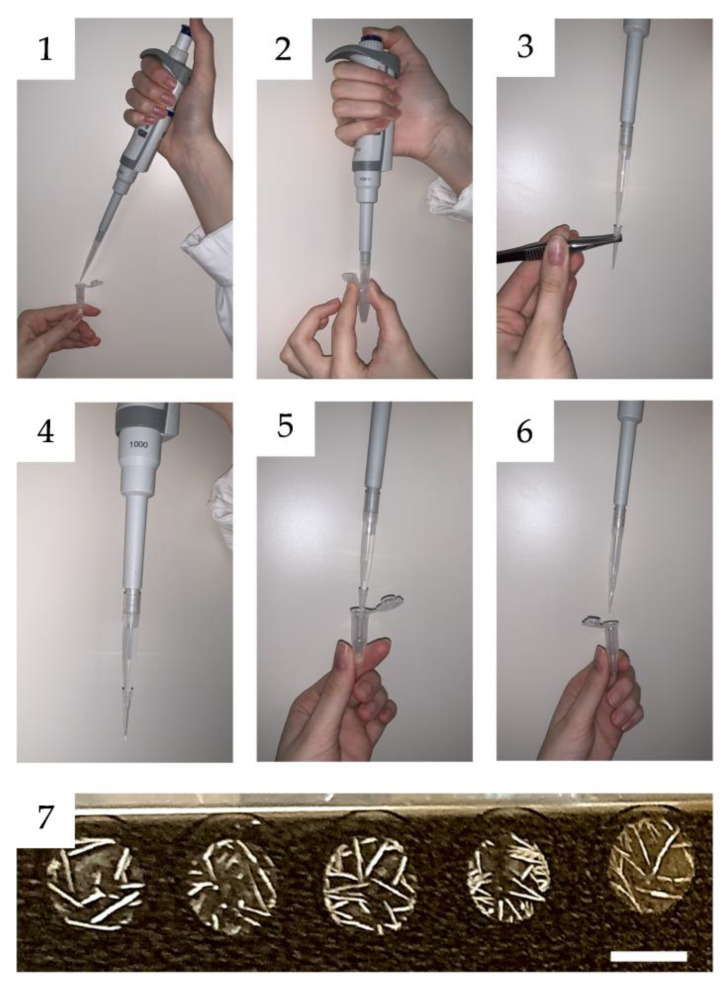
Modified ZipTip protocol for improving cell proteomic footprinting. In total, 1 mL of peptide solution in an Eppendorf tube (**1**) is intended to be analyzed by mass spectrometry to obtain a cell proteomic footprint sampled with an autosampler with a 1 mL pipette tip (**1**,**2**). ZipTip, previously prepared for use according to the manufacturer’s instructions, is connected to the pipette tip (**3**,**4**). The solution is slowly passed through the ZipTip back into the Eppendorf tube (**5**,**6**). The cycle is repeated seven times using the same ZipTip. Then, the ZipTip is washed, and the peptides are eluted according to the manufacturer’s instructions. The eluted peptides are mixed with the DHB matrix and, upon drying, form crystals (**7**), which are convenient for obtaining matrix-assisted laser desorption ionization (MALDI) mass spectra in the manual mode (scale bar: 3 mm).

1. The peptide sample is desalted using ZipTip_C18_ (Millipore Corp., Billerica, MA, USA) according to the manufacturer’s protocol with modifications (Figure 5). The concentration of surface antigens in the solution is incredibly low (for example, 2 μg/mL [103]), and the concentration of individual peptides is many orders of magnitude lower. As a result, the typical ZipTip procedure, which entails filling and withdrawing a 10 μL solution, frequently fails to produce a mass spectrum that is saturated with peptides. Passing 1 mL of the solution through the ZipTip several times increases the amount of peptides that can be caught by the ZipTip.
2. MALDI samples are prepared using a standard ’dried droplet’ method with 2,5-dihydroxybenzoic acid (DHB) as a matrix.
3. Peptide mass spectra are acquired on a MALDI-TOF mass spectrometer (e.g., MicroFLEX, Bruker Daltonik, Germany) in a linear positive ion mode. Mass peak lists are formed manually. The mass spectrometer is set up for the priority detection of ions with an m/z range of 600 to 3500 and a mass accuracy of at least 100 ppm. All peaks above the noise level are selected to generate a cell proteomic footprint [46]. The mass spectra of control samples (Table 3) are used to remove irrelevant masses from the proteomic footprint.
4. The footprint’s peptide masses are binned in 0.2 Da intervals and encoded in a binary format, with 1 representing the presence of a measured peptide mass in an interval and 0 representing its absence. Any suitable software can be used for this.

The reference mass spectra should be obtained under the same conditions and on the same equipment.

The low concentration of peptides, as well as an excess of polymers (e.g., released from culture plates that are usually coated with them), in the solution subjected to mass spectrometry, complicates the acquirement of a high-quality footprint. The use of an established modified method with ZipTip is recommended, which allows a sample with a higher concentration of peptides to be obtained (Figure 5).

The type of mass spectrometry mentioned above is not obligatory. Initial data on cell footprinting are obtained using MALDI-TOF mass spectrometry, which can be considered as approved for CPF. However, instead of MALDI [104], electrospray ionization (ESI) [105] can be used. Instead of sample processing with ZipTip, mass spectrometry may be combined with high-performance liquid chromatography. Before the application of any type of mass spectrometry, test runs should be performed and optimal options for acquiring footprints should be defined.

The use of a DHB matrix and a manual mode of mass spectra acquisition also helps a proper proteomic footprint to be obtained. During the drying of the matrix with the sample on the mass spectrometric target, the peptides crystallize separately from the contaminating polymers (Figure 5), making it possible to obtain more pure mass spectra of the peptides.

### 5.3. Cell Proteomic Footprint Processing

Binary-encoded footprints are submitted for correlation analysis, and calculated Spearman’s correlation coefficients between mass lists are used as a distance matrix. Any suitable software can be used for this (e.g., Matlab, R, SPSS, or Statistica). The use of Euclidian distances instead of Spearman’s correlations is also allowable and gives comparable results. The correlation coefficient scale, on the other hand, provides a more receptive measure of similarity between footprints (scale is always between −1 and 1).

In some cases, if the separation of footprints is challenging, the binary-encoded footprints are submitted for principal component analysis (PCA), and the distances between the projections of footprints on the first (or second) principal component (i.e., scores) are used instead of correlation coefficients [31]. Usually, such an improvement is excessive, but in complex situations, it sufficiently increases the efficacy of CPF. 

**Figure 6:**
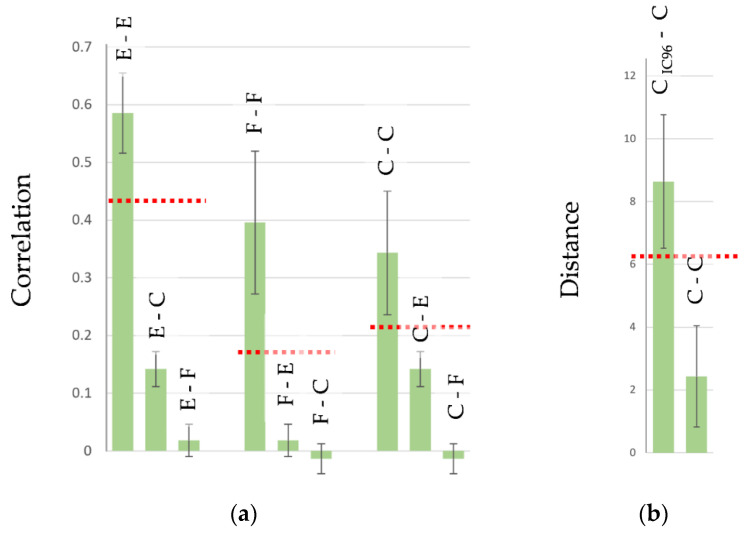

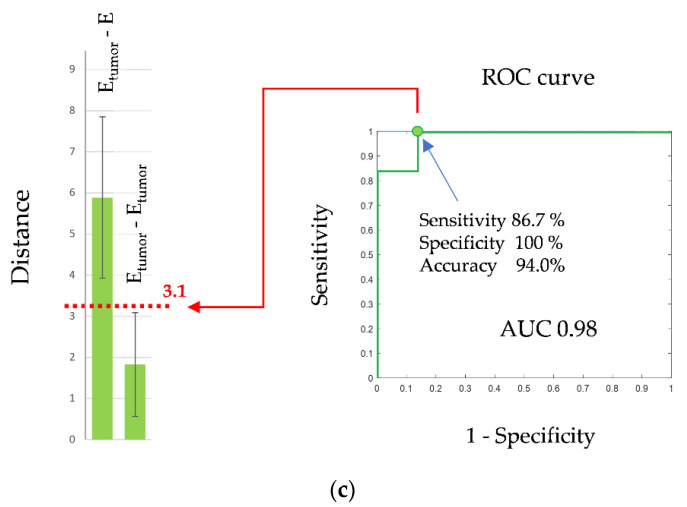
CPF authentication of cell type and subpopulation. (**a**) Cell type authentication. Footprints of endothelial cells correlate with each other (E-E) with higher correlation coefficients than with cancer cells (E-C) and fibroblasts (E-F). The footprints of fibroblasts correlate with each other (F-F) with higher correlation coefficients than with endothelial cells (F-E) and cancer cells (F-C). Footprints are taken from [31,46,80]. (**b**) The authentication of a subpopulation of cancer cells (MCF-7). The distance from the footprints (scores on PC1) of the drug-selected subpopulation (selection with an IC96 dose of etoposide) to other subpopulations of cancer cells is higher (C_IC96_-C) than the distance between cancer cells in the ‘other’ subpopulations (C-C). Footprints are taken from [80]. (**c**) The authentication of a subpopulation of endothelial cells (HMECs). The distance between the footprints (scores on PC2) of endothelial cells with the tumor-induced phenotype (E_tumor_-E_tumor_) is lower than the distance from them to other subpopulations of endothelial cells (E_tumor_-E). Footprints are taken from [31]. An ROC curve is used to find the optimal threshold value (red dotted lines) to separate footprints according to the cell type and subpopulation. The provided data are consistent with the data presented in Table 1 and Table 2.

If the footprint of the tested cell preparation falls into a group of reference cells, the CPF test for cell authentication is passed [46]. This confirms that the tested cells are produced as required, and their composition is suitable for use. The threshold between reference and control groups can be visually determined (when the reference group is distant from the control group(s)). To objectively define the threshold, a point on an ROC curve [104] corresponding to the highest accuracy in separating the reference group from the control group(s) can be used (Figure 6). To build an ROC curve, any software providing such an option can be used (e.g., Matlab, R, SPSS, Statistica, etc.).

## 6. Supplement to Quality Control

Any cell product used for clinical trials or sales on the market undergoes quality assessment, both at all stages of production and at its release. This is realized through the use of quality control (QC) in the production process [104,106]. As a rule, QC includes methods used for detecting potential infectious agents, as well as assessing the level of excipients and adventitious agents that can be adsorbed on the cells and that are undesirable to be present in the final product. The description of the QC program, which has different characteristics for each cell product, is beyond the scope of this review. However, the reviewed studies allow the suggestion of a supplementary QC program (Table 4) in order to include CPF in the production of cell products.

## 7. CPF Implementation Guide

In order to develop cell products, it is recommended that the existence of CPF is taken into account, especially when it comes to the development and manufacturing of cell-based cancer vaccines. Even if there are doubts about the practicality of using CPF, the analysis of footprints by the use of annotated program source code provided in the Supplementary Materials are recommended. Using them, the footprint analysis results presented in the review can be quickly and easily obtained. In this way, the scale of the problem of heterogeneity among cell subpopulations can be felt, and researchers can quickly acquire the necessary skills to work with footprints. With such experience, an informed decision on the appropriateness of the use of CPF at work can be made.

## 8. Final Remarks

From the possible directions for further research on CPF technology, one can single out the study of cell quality in 3D culture, organoids, and freshly isolated cells. It is assumed that the cells in these approaches have a molecular phenotype closer to native cells than cells cultured in a monolayer. The use of CPF to characterize them and reveal (or confirm) useful properties seems interesting. 

Although trypsin is the most common protease in proteomics, other proteases are also used that allow proteins to be cleaved at sites other than those of trypsin. Therefore, the simultaneous use of different proteases generates peptide fingerprints rich in various peptides, which improves protein analysis. If strengthening the CPF test is necessary for identifying extremely similar cell subpopulations, the same approach can be tried. 

## 9. Conclusions

Scientific data accumulated to date show that CPF may supplement the cell-based development and manufacture of immunotherapeutics, making them state-of-the-art. Cancer cells used to produce vaccines must be checked to assess the consistency of antigenic profiles with target cells, allowing the vaccines to be clinically effective. Cancer cells that were not authenticated by CPF cannot be considered suitable for vaccination, since only genome-level authenticated cells may refer to a variety of subpopulations characterized by a different molecular phenotype, including the lack of therapeutic properties. The data presented in the review on the authentication of therapeutically efficient subpopulations make it possible to integrate the CPF test into the routine of laboratories involved in the design of vaccines, as well as into the QC program of their manufacture.

## Figures and Tables

**Table 1:** The separation of different cell types based on CPF (proof-of-concept of high specificity of cell proteomic footprints at cell type level).

Cells	Sensitivity (%)	Specificity (%)	Accuracy (%)	AUC
ECs versus non-ECs ^1^	100.0	100.0	100.0	1.00
Cancer cells versus non-cancer cells ^2^	100.0	96.4	96.7	0.998
Fibroblasts versus non-fibroblasts ^3^	98.9	98.8	98.9	0.9996

^1^ ECs, primary cultures (*n* = 8) of human microvascular endothelial cells (HMECs); non-ECs: fibroblast cell lines with different origins (*n* = 28) and cancer cell lines (*n* = 8). ^2^ Cancer cells (*n* = 8) and non-cancer cells: fibroblast cell lines with different origins (*n* = 28) and ECs (*n* = 8). ^3^ Fibroblast cell lines with different origins (*n* = 28) and non-fibroblasts: cancer cell lines (*n* = 8) and ECs (*n* = 8). Sensitivity—the percentage of correctly identified positive results (tested cells are correctly assigned to a specific cell type); specificity—the percentage of correctly identified negative results (tested cells correctly do not assigned to specific cell type); accuracy—the percentage of correctly identified positive and negative results. The AUC is the area under the receiver operating characteristic (ROC) curve. The ROC reflects the dependence sensitivity from the specificity. The closer the AUC is to 1, the more reliable the test is. Footprints are taken from [31,46,80].

**Table 2:** The separation efficiency of different cell subpopulations based on CPF.

Cells Subpopulations	Sensitivity (%)	Specificity (%)	Accuracy (%)	AUC
Cancer cells [IC96^Etop^, IC96^Etop twice^] versus [Untr, IC50^Etop^, IC50^Etop twice^, IC96^Etop^, IC96^Dox^, IC96^Tmx^] ^1,2^	100.0	90.0	95.0	0.99
ECs versus tumor-stimulated ECs ^3^	86.7	100.0	94.0	0.98
Adipose fibroblasts versus skin fibroblasts	100.0	100.0	100.0	1.00
Skin fibroblasts versus dermal papilla fibroblasts	94.4	66.7	68.8	0.87
Dermal papilla fibroblasts versus adipose fibroblasts	83.3	83.3	83.3	0.89

^1^ ‘Untr’, untreated MCF-7 cells; IC96^Etop^, MCF-7 cells treated with an IC96 dose of etoposide; IC96^Etop twice^, MCF-7 cells treated with two separate IC96 doses of etoposide; IC96^Tmx^, MCF-7 cells treated with an IC96 dose of tamoxifen; IC96^Dox^, MCF-7 cells treated with an IC96 dose of doxorubicin; IC50^Etop^, MCF-7 cells treated with an IC50 dose of etoposide, IC50^Etop twice^; MCF-7 cells treated with two separate IC50 doses of etoposide. ^2^ PCA was applied to improve the separation footprints (scores for PC1 were taken to calculate the distances between footprints). ^3^ PCA was applied to improve the separation of footprints (scores for PC2 were taken to calculate the distance between footprints). ECs, human microvascular endothelial cells (HMECs). Sensitivity—the percentage of correctly identified positive results (tested cells are correctly assigned to a specific cell type); specificity—the percentage of correctly identified negative results (tested cells correctly do not assigned to specific cell type); accuracy—the percentage of correctly identified positive and negative results. The AUC is the area under the ROC curve. The ROC reflects the dependence sensitivity from the specificity. The closer the AUC is to 1, the more reliable the test is. Footprints are taken from [31,46,80].

**Table 3:** A list of control samples required for CPF.

#	Control Sample	Data Provided by the Mass Spectrum of the Sample
1	Blank (mass spectrum of DHB matrix with HBSS and without any peptides).	A list of irrelevant mass peaks (from solution and DHB matrix) that are subtracted from the cell proteomic footprints.
2	Solution of proteomics-grade trypsin (concentration of 0.2 μg/mL in HBSS; the sample is incubated for 20 min at 37 °C and a minimum volume of 10% acetic acid is added to acidify the solution and terminate the action of trypsin).	A list of mass peaks related to trypsin (trypsin autolysis products). The list allows for the detection of trypsin peaks in the mass spectrum to remove them from the proteomic footprint. Well-known peptides related to autolysis products can be used for the calibration of mass spectra. This list of mass peaks is obtained for each used lot of trypsin preparation.
3	Culture medium with supplements (e.g., FBS and/or other supplements).	A list of mass peaks related to culture medium with FBS. The list of mass peaks is obtained once for each FBS lot used.
4	Proteomics-grade trypsin (0.2 μg/mL) incubated in a culture medium with supplements (20 min, 37 °C).	A list of mass peaks related to proteolytic peptides from culture medium supplements (e.g., FBS). The list of mass peaks is obtained once for each FBS lot used.
5	Cells were frozen and thawed three times before being centrifuged at 600 g for five minutes.	A list of mass peaks related to sample contamination with intracellular content. The list is obtained once for each cell’s lot.
6	Sample #5 was incubated with proteomics-grade trypsin (20 min, 37 °C; trypsin 0.2 μg/mL).	A list of mass peaks related to sample contamination with intracellular content. The list is obtained once for each cell’s lot.

FBS, fetal bovine serum; DHB, 2,5-dihydroxybenzoic acid; HBSS, Hanks’ balanced salt solution.

**Table 4:** Supplement to the quality control (QC) program to include cell proteomic footprinting (CPF) in the manufacture of cell-based immunotherapeutics (vaccines based on whole cells, cell lysates, cell membranes, and antigenic essences).

QC Test	Failed Specification	Sources of Variation	Impact of Variation on the Process and Product Attributes	Control of the Variation
Cell authentication using CPF. The test can be used after isolating the cells (if applicable), after establishing cell culture (if applicable), after initiating cell culture from a cell bank, while cultivating the cells, and during final product release testing.	Cell death during treatment with trypsin (more than 1%).	Cell damage via trypsin and/or fluid shearing. The sample for CPF is contaminated with intracellular content.	Leads to a cell proteomic footprint contaminated with intracellular content. CPF results are not accepted. The cell product cannot be qualified for use.	1. To exclude cell damage that occurs as a result of protease treatment, another lot of trypsin should be used. If no effect is observed, trypsin can be used with a higher activity than that used (with a proportional dilution). 2. To minimize cell destruction by fluid shearing, careful cell manipulation can be used and the protocol can be optimized accordingly. As a last resort, the use of cytoprotective agents can be considered at the cell wash stage to decrease cell damage [38]. Importantly, cytoprotectants are polymers; therefore, they should not contaminate the analyzed trypsin solution, as they will interfere with peptide peaks in the mass spectrum.
	Authentication of cells based on CPF results.	The misidentification of cells.	Cell-based products cannot be produced.	1. Cells must be disposed of, and their storage and incubation sites (hoods), manipulation sites (laminar systems), and reusable instruments must be sterilized. 2. The cell culture must be recovered from stock in the master or the working cell bank. 3. The possible contact of cells with other cell types at the manufacturing site should be avoided. 4. The cell culture process should be rechecked to reduce the risk the cells becoming recontaminated.
		Cross-contamination of cells with other mammalian cells.	Leads to less purity in the cell-based product. In the case of a cancer vaccine, the vaccine becomes unusable due to the increased content of non-target antigens.	
		CPF is carried out incorrectly.	The product cannot be qualified for use.	The source of variation should be defined in the sample preparation, mass spectrometry, and data treatment of CPF. For this, the control samples (Table 3) should be used.
		The sample for CPF is contaminated by peptides that originate from culture medium proteins (e.g., from fetal bovine serum).	Cells are not qualified for producing cell products.	1. The source of variation should be verified using the control mass spectra (Table 3). 2. When the sample is contaminated with peptides that originate from culture medium (e.g., from fetal bovine serum), improved cell washing with Hanks’ balanced salt solution (HBSS) is required before the cells are treated with trypsin. If the problem persists, the addition of one more HBSS wash cycle should be considered.
		Cells do not have the required a molecular phenotype.	Cells are not qualified for producing cell products.	1. Cells should be disposed of, or, if possible, the required molecular phenotype should be induced. 2. The manufacturing process should be adjusted to produce cells with the required molecular phenotype.

## Data Availability

Not applicable.

## Supplementary Materials

The following supporting information can be downloaded at: https://www.mdpi.com/article/10.3390/pharmaceutics15020661/s1, aligned_footprints.mat: Matlab matrix with aligned binary-encoded footprints; cell_proteomic_footprints_analysis.m: Matlab source code used for footprint processing.
